# Supplementary material for: Multicow pose estimation based on keypoint extraction
Source: PLoS One. 2022 Jun 3;17(6):e0269259. doi: 10.1371/journal.pone.0269259 (PMC9165835; doi:10.1371/journal.pone.0269259)
Supplement: Attachment — Submitted filename: Response to Reviewers.docx [file pone.0269259.s001.docx]

Dear Editor:

Thank you very much for giving us an opportunity to revise our manuscript, we appreciate editor very much for the positive and constructive comments and suggestions on our manuscript entitled “Multicow Pose Estimation Based on Keypoint Extraction” (Original manuscript number: PONE-D-21-40261). We have studied editor’s comments carefully and have tried our best to revise our manuscript according to the comments. The main corrections and the responds to the editor’s comments are as flowing:

**Responds to the editor’s comments:**

1. **We note that you state that the manuscript was copyedited by AJC. However, we do not feel that the language is sufficiently improved, and as such, we request that you further copyedit the manuscript before it can proceed.**

**Response:**  With the help of American Journal Experts (AJE), the English grammar and usage in the manuscript have been revised again. We believe that the English grammar and usage in the manuscript has been greatly improved. We strongly believe the contribution of manuscript warrants its publication in Plos One. Editing certificate of English grammar and usage was submitted in the attachment.

Thank you and best regards.

Yours sincerely,

Caili Gong

Corresponding author:

Name: Yong Zhang

E-mail: yongz@imau.edu.cn.
