# Supplementary material for: Multicow pose estimation based on keypoint extraction
Source: PLoS One. 2022 Jun 3;17(6):e0269259. doi: 10.1371/journal.pone.0269259 (PMC9165835; doi:10.1371/journal.pone.0269259)
Supplement: Attachment — Submitted filename: Response to Reviewers.docx [file pone.0269259.s002.docx]

Dear Editor and Reviewer:

Thank you very much for your constructive comments and valuable suggestions. We have carefully considered the suggestion of Editor and Reviewer. We have tried our best to improve and made some changes in the manuscript according to your kind advices and detailed suggestions. The changes and corrections will be marked in red in the manuscript. This document summarizes our revisions and responses in the following. Thank you again for all your help with our manuscript.

**Comments from Editors**

**Responds to the editors' comments:**

1.Please ensure that your manuscript meets PLOS ONE's style requirements, including those for file naming. The PLOS ONE style templates can be found at

https://journals.plos.org/plosone/s/file?id=wjVg/PLOSOne_formatting_sample_main_body.pdf and

<https://journals.plos.org/plosone/s/file?id=ba62/PLOSOne_formatting_sample_title_authors_affiliations.pdf>

**Responds:** Thank you very much for the kind reminder. We have downloaded the LaTeX template from PLOS ONE's official website and read the LaTeX guidelines. We have revised some of the formatting and will do our best to make our manuscript conform to PLOS ONE's style.

2. We note that the grant information you provided in the ‘Funding Information’ and ‘Financial Disclosure’ sections do not match.

When you resubmit, please ensure that you provide the correct grant numbers for the awards you received for your study in the ‘Funding Information’ section.

**Responds:** We are very sorry for the mismatch of grant information that was provided in the ‘Funding Information’ and ‘Financial Disclosure’ sections. We have revised the grant information and do our best to provide the correct grant numbers for the awards in the ‘Funding Information’ section.

3. Thank you for stating the following financial disclosure:

“NO, the funders had no role in study design, data collection and analysis, decision to publish, or preparation of the manuscript.

At this time, please address the following queries:

a)        Please clarify the sources of funding (financial or material support) for your study. List the grants or organizations that supported your study, including funding received from your institution.

b)        State what role the funders took in the study. If the funders had no role in your study, please state: “The funders had no role in study design, data collection and analysis, decision to publish, or preparation of the manuscript.”

c)        If any authors received a salary from any of your funders, please state which authors and which funders.

d)        If you did not receive any funding for this study, please state: “The authors received no specific funding for this work.”

Please include your amended statements within your cover letter; we will change the online submission form on your behalf.

**Responds:** We are very sorry for missing the funding information.We have listed the grants that supported our study in the ‘Funding Information’ section. This study was funded by the National Natural Science Foundation of China under Grant 61966026, Grant 62161034 and Grant 61561037. The funders had no role in study design, data collection and analysis, decision to publish, or preparation of the manuscript. The authors appreciate the funding organization for their financial supports.

4. In your Data Availability statement, you have not specified where the minimal data set underlying the results described in your manuscript can be found. PLOS defines a study's minimal data set as the underlying data used to reach the conclusions drawn in the manuscript and any additional data required to replicate the reported study findings in their entirety. All PLOS journals require that the minimal data set be made fully available. For more information about our data policy, please see http://journals.plos.org/plosone/s/data-availability.

Upon re-submitting your revised manuscript, please upload your study’s minimal underlying data set as either Supporting Information files or to a stable, public repository and include the relevant URLs, DOIs, or accession numbers within your revised cover letter. For a list of acceptable repositories, please see http://journals.plos.org/plosone/s/data-availability#loc-recommended-repositories. Any potentially identifying patient information must be fully anonymized.

Important: If there are ethical or legal restrictions to sharing your data publicly, please explain these restrictions in detail. Please see our guidelines for more information on what we consider unacceptable restrictions to publicly sharing data: http://journals.plos.org/plosone/s/data-availability#loc-unacceptable-data-access-restrictions. Note that it is not acceptable for the authors to be the sole named individuals responsible for ensuring data access.

We will update your Data Availability statement to reflect the information you provide in your cover letter.

**Responds:** We are very sorry for our negligence of the links to images datasets. The minimal data set underlying the results described in our manuscript can be found at <https://www.kaggle.com/twisdu/dairy-cow>. All data in our manuscript will be fully shared without restriction. The link to the minimal dataset has been added in the Supporting Information files when submitting the revised manuscript.

5. PLOS requires an ORCID iD for the corresponding author in Editorial Manager on papers submitted after December 6th, 2016. Please ensure that you have an ORCID iD and that it is validated in Editorial Manager. To do this, go to ‘Update my Information’ (in the upper left-hand corner of the main menu), and click on the Fetch/Validate link next to the ORCID field. This will take you to the ORCID site and allow you to create a new iD or authenticate a pre-existing iD in Editorial Manager. Please see the following video for instructions on linking an ORCID iD to your Editorial Manager account: <https://www.youtube.com/watch?v=_xcclfuvtxQ>

**Responds:** Thank you very much for the kind reminder. The corresponding author have an ORCID iD and that it is validated in Editorial Manager. And we have updated the Information in PLOS ONE Editorial Manager Submission System.

**Comments from Reviewers:**

Reviewer #1: The article presents an application of current detection methods to the poses of cows on a farm. The experiments have been explained and performed according to the field’s standard. A couple of points remain, that need attention before publication in my opinion.

**Responds to the reviewer's comments:**

1) Data availability
• additional information in the beginning of the PDF states, that all relevant data is freely available and that it is available within the manuscript and its Supporting Information files.
• But the manuscript does not contain any links to images, videos or code.

**Responds:**

We are very sorry for our negligence of the links to images datasets. The minimal data set underlying the results described in our manuscript can be found at <https://www.kaggle.com/twisdu/dairy-cow>. All data in our manuscript will be fully shared without restriction. The link to the minimal dataset has been added in the Supporting Information files when submitting the revised manuscript.

2) Ethics
   • IACUC and approval number were not mentioned
   ◦ it was also not mentioned, that those were not necessary. Please state why the relevant authorities do not consider this an animal experiment, that needs to be approved. Or if they do, state the approval number.

• How did non-contact machine vision improve animal welfare during recording?
◦ As stated under “data acquisition”
◦ perhaps you meant, that the research could in the future improve welfare?

**Responds:**

Thank you very much for your kindness suggestion. This study was carried out at Inner Mongolia Flag Animal Husbandry Co., Ltd. Inner Mongolia Agricultural University has conducted scientific research with Inner Mongolia Flag Animal Husbandry Co., Ltd. in Hohhot for more than five years. In the stage of experiment, the data was taken with the consent and direction of the company. The study does not require approval from any relevant authorities.

The traditional way based on manual observation methods to detect the behavior of dairy cows increases the probability of human-animal contact, which can lead to some zoonotic diseases. The way of wearable sensors to collect the behavior of dairy cows seriously interferes with the cows during the installation of the equipment, which is likely to cause a strong stress response to the dairy cows. The data in this paper was acquired by the monitoring camera, which was fixed on the fence of the breeding site at a height of 4 m. During the data collection process, neither the collection equipment nor the experimenter contacted the cows. It did not interfere with the normal activities of the cows, and did not cause the cows' stress response. Compared with traditional manual inspection and using wearable sensors, it can realize continuously monitoring the activity and health of dairy cows without contact. If the project could be implemented in the future, it can complete non-contact animal behavior monitoring and improve animal welfare. According to your kind suggestion, we have revised the detailed description of data acquisition in the manuscript

3) Funding
• where was funding coming from? If the authors did not receive funding, it has to be stated

**Responds:**

We are very sorry for missing the funding information. This research was funded by the National Natural Science Foundation of China under Grant 61966026, Grant 62161034 and Grant 61561037. The funders had no role in study design, data collection and analysis, decision to publish, or preparation of the manuscript. We have corrected the funding information in the ‘Funding Information’ section when submitting our revised manuscript.

4) Data
• LabelImg was used, please give a source or citation
• Mosaic enhancement
◦ why did you use it? Why not other augmentation methods like mirroring or rotation?
◦ Should it not be called “mosaic augmentation” instead of “enhancement”
• How were the data set frames picked? It cannot be random from the videos, since you have exactly 600 images for each condition (standing, walking, lying). How exactly were validation and test set chosen?
• How often do the cows occlude each other by more than 50%? You state, that it is rather seldom in a real farm environment. Could you estimate how much it happens in your data?

**Responds:**

Thank you very much for your detailed suggestion.

LabelImg: The source of LabelImg annotation tool is <https://github.com/tzutalin/labelImg>. We have added the source citation in the revised manuscript.

Mosaic augmentation: As your kind suggestion, we revised the expression to Mosaic augmentation. It is a kind of data augmentation method. It has the advantage of enriching the background of the detection content. The data of 4 images can be calculated at a time during Batch Normalization (BN) calculation. The 4 images were mirrored, flipped, cropped before they were stitched together.

Data set: The data frames were manually selected so that the selected images contain each condition (standing, walking, lying). The validation and test sets are randomly drawn from the overall dataset.

Occlusion: During the experiment, we adjusted the height of the camera to make the cow objects less occluded in video. To test the robustness of cow occlusion effects on detection accuracy, we lowered the shooting height in some video. When the little cow body was occluded by more than 50%, the detection accuracy significantly decreased. As your kind suggestion, we will continue to study the occlusion situation to improve the detection accuracy and meet the needs of large-scale precision farming.

5) Performance
• The abstract states, that the algorithm exhibited a higher detection rate. Higher than what?
• Table 1
◦ In the text and the table it says “IOU” in places, where in my understanding “IOU threshold” is meant. In case I misunderstand, please make it more clear, what “ average precision when the IOU is equal to 0.5” means. If it should indeed be “IOU threshold”, please correct.
◦ What is the difference between AP and AP50? They both appear in the rows, where the IOU threshold is 0.5. This confusion might be connected to the previous point.
◦ What are the “400 images tested simultaneously”?

**Responds:**

Thank you very much for your constructive comments and valuable suggestions. As your kind suggestion, we revised the expression in manuscript. This study combined YOLO v4 object detection and pose estimation to classify daily behaviors of multi-cow. The Test result showed that the detection accuracy was higher than that in previous research results. Song et al. [6] proposed a skeleton extraction model of cows in walking states with a high accuracy rate of up to 93.40% when the OKS was 0.75. Hahn Klimroth et al. [22] presented a multistep convolutional neural network for detecting three typical poses of African ungulates, obtaining a high accuracy of 93%. Chen et al. [24] proposed an algorithm based on YOLACT with high detection speed and accuracy for real-time detection and tracking of multiple parts of pig bodies. The detect accuracy of the algorithm in the data set could reach up to 90%,

In Table 1, IoU means the Intersection over Union, and IoU threshold is a judgment threshold. If the IoU of the predicted box and the ground truth is greater than or equal to the IoU threshold, the predicted result is considered to be TP; otherwise, the predicted result is considered to be FP. When IoU is equal to 0, the prediction result is considered to be FN. AP50 is the average precision when the IoU threshold is set to 0.5. AP is the mean value when the value of IOU is taken from 0.5 to 0.95 and the step size is 0.05.

The 400 images refer to the image test set containing all four cases listed in the table.

6) Structure
• In the methods section the model setup is detailed. State more clearly what parts are done as in yolo v4 and what you have inserted yourself.

**Responds:**

Thank you very much for your constructive comments and valuable suggestions. Considering the Reviewer’s suggestion, we have added the detailed statement of the improvement YOLO v4. Firstly, the original loss function consists of three parts, namely the bounding box regression loss, the confidence loss and the category loss. In this paper, we only study on the cow objects, category loss does not need to be considered. We modified the overall loss function of the object detection network into confidence loss and regression loss. Confidence loss was used to describe whether there was an object in the grid cell by calculating the binary cross entropy. Regression loss was used to describe the position and size difference between the annotated object and the predict object. Then, we adopted the transfer learning in this paper. We used the pretrained network weights on the PASCAL VOC 2012 dataset and initialized the network model. And then the network was fine-tuned by self-built dataset to achieve better training effect. We have revised the presentation of this section in the manuscript.

7) Conclusion
• It is necessary to assess at least roughly, whether the performance of the system would be adequate for applications in the area such as estrus detection. To make a statement under some assumptions in the conclusion would already be good. This would put the numbers into a useful context.

**Responds:**

Thank you very much for your valuable suggestions. When a cow is in lameness, estrus and before calving, the pose will change frequently. The pose estimation and behavior classification methods of dairy cow based on skeleton feature extraction in this study have certain reference significance for animal behavior researchers. And this study can provide further data support for lameness detection, estrus detection and the prediction of calving in large-scale precision farming. We will focus on improving the real-time performance while ensuring high detection accuracy and reducing the number of parameters and calculations in the following work.
